# Minimally acceptable criteria and required sample size for an accuracy study of non-invasive prenatal testing of sickle cell disease in screen positive women in England: results of a decision tree model

**DOI:** 10.1186/s41512-025-00192-w

**Published:** 2026-06-15

**Authors:** Vittoria Vardanega, Ania Bobrowska, Benjamin Ruban-Fell, James A. Doorbar, Silvia Lombardo, Farah Seedat, Yvonne Daniel, Amanda Hogan, Mariska M. G. Leeflang, Kathy Mann, Anna Schuh, John Marshall

**Affiliations:** 1 Costello Medical, Cambridge, UK; 2 UK National Screening Committee, Department of Health and Social Care, London, UK; 3 Institute of Infection and Immunity, St. George’s University of London, London, UK; 4 Special Haematology Department, Guy’s and St, Thomas’ Hospital, London, UK; 5 NHS Sickle Cell and, Thalassaemia Screening Programme, London, UK; 6 Department of Epidemiology and Data Science, Amsterdam University Medical Centers, University of Amsterdam, Amsterdam, The Netherlands; 7 East Genomic Laboratory Hub, Cambridge University Hospitals NHS Foundation Trust, Cambridge, UK; 8 Department of Oncology, University of Oxford, Oxford, UK

**Keywords:** Sickle cell disease, Decision tree model, Screening, Minimally acceptable criteria, Sample size calculation, Prenatal, NIPT, Non-invasive prenatal testing

## Abstract

**Background:**

The screening pathway for sickle cell disease (SCD) in England starts with a carrier status blood test for the pregnant woman. Following a positive result, the test is offered to the biological father. Where both results are positive, further invasive testing is offered to assess the risk of SCD to the fetus. When the father is unavailable, the timely offer of further testing may be delayed and even missed, leaving the pregnant woman underinformed and limiting reproductive choice. Non-invasive prenatal testing (NIPT) presents an alternative to paternal testing and diagnostic accuracy studies of NIPT in SCD screening are required to inform utility and application. We explored the minimally acceptable sensitivity and specificity to inform such an accuracy study of NIPT.

**Methods:**

A decision tree model was produced to identify the minimally acceptable sensitivity and specificity of NIPT. Stakeholder engagement identified a positive predictive value (PPV) equal to the paternal carrier blood test and two sensitivity/specificity scenarios: Scenario 1 should result in < 10 false negative diagnoses per year; Scenario 2 should result in ≤ 2 false negative diagnoses per year. Subsequently, the minimally acceptable sensitivity and specificity were used to calculate the sample size for a hypothetical accuracy study of NIPT in each scenario.

**Results:**

Scenario 1 led to a minimally acceptable sensitivity of 96.0% and specificity of 88.5% and corresponding negative predictive value (NPV) of 99.81%, PPV of 25.51% and accuracy of 88.80%. Utilising an expected prevalence of SCD of 3.94%, the resulting sample size would include 315,824 total pregnancies.

Scenario 2 led to a minimally acceptable sensitivity of 99.0% and specificity of 88.0% and corresponding NPV of 99.95%, PPV of 25.29% and accuracy of 88.43%. The resulting sample size would include 65,509 overall pregnancies.

**Conclusions:**

While the sample size calculated for each scenario is unfeasible if considering a prospective cohort study, approaches are presented to achieve realistic sample sizes. Increasing the sensitivity and specificity levels might be considered if this is likely to be achievable based on available studies, albeit limited in volume. Consideration could be given to study design mitigations that are consistent with guidance on accuracy studies in low prevalence settings.

**Supplementary Information:**

The online version contains supplementary material available at 10.1186/s41512-025-00192-w.

## Background

### Current practice

Sickle cell disease (SCD) is a genetic condition characterised by chronic anaemia, acute painful episodes, organ infarction, chronic organ damage and a significant reduction in life expectancy [1]. It is estimated that there are over 15,000 people living with SCD in the United Kingdom (UK), with the condition being more common among those from African and Caribbean backgrounds [2, 3].

SCD screening is one of 11 National Health Service (NHS) national population screening programmes available in England recommended by the UK National Screening Committee (NSC) [4–6]. Screening for SCD involves an initial blood test (herein referred to as the maternal carrier blood test) offered to all pregnant women living in areas of high SCD prevalence [7]. This is a full blood count and further analysis using high performance liquid chromatography (HPLC) or capillary electrophoresis (CE) techniques [7]. In areas of lower SCD prevalence, full blood count results and the Family Origin Questionnaire are used to determine the risk of carrying SCD, wherein pregnant women deemed to be at high-risk have their blood samples analysed using HPLC/CE [7]. If the pregnant woman receives a positive maternal carrier blood test result, indicating that she is a carrier of a gene mutation which causes SCD, the biological father is offered the blood test (herein referred to as the paternal carrier blood test) to then determine the risk to the fetus [7]. Data from the SCD screening pathway in England has shown the uptake of the paternal carrier blood test to be 70.4%, compared with 99.87% for pregnant women offered the maternal carrier blood test in the screening year 2019 to 2020 [8]. When both maternal and paternal carrier blood test results are available, only cases where both are positive for a sickle cell allele will be offered invasive prenatal diagnosis (PND). However, when the pregnant woman has a screen-positive carrier blood test and the biological father is unwilling or unavailable for testing, this lack of risk information means that she will be offered PND as the next step in the screening pathway. This group made up 24% of all screen-positive women in the 2019 to 2020 screening year [8].

In the current standard of care, PND consists of chorionic villus sampling or amniocentesis. Despite high risks often being quoted to women, a comprehensive meta-analysis has shown that when performed by experienced operators, the risk of miscarriage following these procedures is negligible compared to control groups with similar risk profiles that had not undergone the procedure [9]. However, even if the risk is negligible, the invasive nature of the test is likely to cause discomfort in the women to whom it is offered, potentially contributing to the decision to decline the test offer.

Many women in England decline the offer of PND for SCD, with women more likely to decline the test once their pregnancy has progressed to the second trimester [8]. This may be due to absence of the paternal carrier blood test result, timing of the offer following a protracted attempt to identify and test the biological father, personal values, or hesitance about the invasive procedure. The outcome of these pregnancies remains uncertain until dried bloodspot screening is offered in the neonatal period.

### Non-invasive prenatal testing

Non-invasive prenatal testing (NIPT) a type of cell-free DNA (cfDNA) testing presents a possible future alternative (in the form of non-invasive prenatal diagnosis; NIPD) to chorionic villus sampling or amniocentesis. NIPT has been successfully applied to prenatal screening and prenatal diagnosis of both chromosomal and monogenic conditions, though the test is particularly challenging for autosomal recessive conditions. NIPT involves the analysis of DNA circulating in the maternal bloodstream, and small studies have suggested it may be effective in identifying pregnancies at risk of SCD [10, 11]. However, NIPT has not yet been proven to be accurate enough to be used as NIPD and therefore should not currently be considered as a replacement for PND methods. The usefulness of NIPT, at this stage, may therefore lie in its application as a screening test to replace the paternal carrier blood test, whether or not the biological father is available for testing. This has been explored in a parallel publication, “Modelling the Cost-Effectiveness of Non-invasive Prenatal Testing in the English Sickle Cell and Thalassaemia Screening Pathway” [12]. Including NIPT in the screening pathway before the offer of PND could provide more accurate risk information to more women to inform discussions regarding PND, in particular, improving equity for the 24% of women with a positive maternal carrier blood test in the absense of the paternal carrier blood test result [8]. Eliminating the workload associated with inviting and testing the biological father may also improve efficiency within the health service.

Finally, depending on the negative predictive value (NPV) of the future NIPT, there could be a significant reduction in the number of women undergoing invasive PND when the fetus is unaffected. As NIPT is a screening test, those with screen-positive results would continue to be offered PND.

Therefore, a diagnostic accuracy study investigating the accuracy of NIPT is needed to robustly assess whether it represents a suitable alternative to the paternal carrier blood test in the SCD screening pathway in women with positive carrier blood tests. The present analysis aims to support the design of such study, by identifying the minimally acceptable sensitivity and specificity to ensure NIPT efficacy and to inform the sample size required.

## Methods

### Study approach

**Fig. 1:**
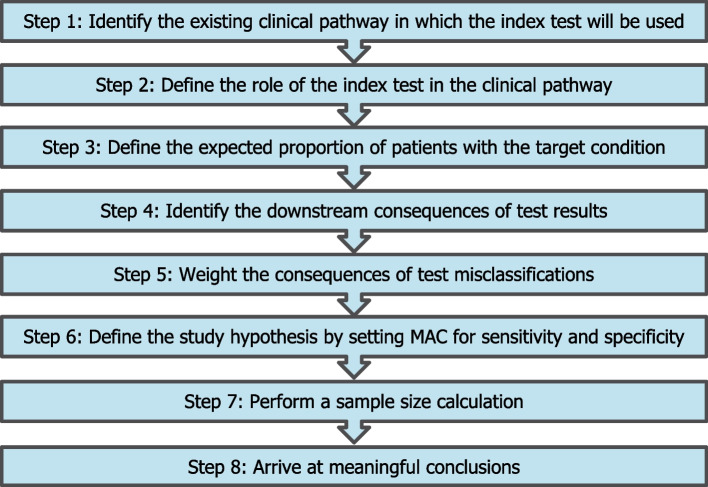
The step-wise approach outlined in Korevaar et al. 2019. Abbreviations: MAC; minimally acceptable criteria. Source: Korevaar et al. 2019 [13]. Minor formatting changes have been made. Permission given for reuse under the terms of the Creative Commons Attribution 4.0 International License, available at: http://creativecommons.org/licenses/by/4.0

The overall methodology followed for this study is informed by the approach outlined in Korevaar et al. (Fig. 1) [13], which aims to support researchers in the design of diagnostic accuracy studies. According to this step-wise methodology, the downstream consequences of the test results are identified, and the consequences of test misclassifications are weighted. This allows for the identification of the minimally acceptable criteria (MAC) that the test must fulfil, in terms of sensitivity and specificity, to be considered beneficial for the particular diagnostic scenario. On the basis of the identified MAC, the meaningful hypotheses for the study are defined, and the necessary study sample size is calculated. The Korevaar et al. approach was selected for this study due to its systematic methodology, which in part focuses on quantifying the downstream consequences of a modelled test, and for its replicability [13].

To complete steps 1 to 5 of the Korevaar et al. approach (see Fig. 1 for each step) [13], a decision tree model was developed to rigorously quantify and weigh the downstream consequences of introducing NIPT in the England SCD antenatal screening pathway (see “ Model structure” section). Two workshops with stakeholders from the UK NSC and NHS Sickle Cell and Thalassaemia Screening Programme, experts in the field of cfDNA testing, and test accuracy study experts were held to provide guidance at different stages of the study. During the workshops, relevant information about the study’s objectives, scope, and methodology was shared with stakeholders, ensuring they had a clear understanding of the study and their role. During the first workshop, the modelling approach and model inputs were presented and discussed with stakeholders to ensure they were adequate for application to the screening pathway. The model was then developed based on the feedback received. During the second workshop, the modelled NIPT outcomes and the impact of varying NIPT sensitivity and specificity on these outcomes were presented and discussed, in line with step 6 of the Korevaar et al. approach [13]. Following input from stakeholders on key aspects of the MAC in the second workshop, the required MAC was identified. Additional discussions with experts during the course of manuscript development contributed to the final refinement of the strategy and approach utilized.

Finally, using the prevalence of SCD in the population of women who are screen positive for the carrier blood test, a sample size calculation was performed using the MAC to derive the total number of pregnancies required for a future diagnostic study assessing the accuracy of NIPT, as outlined in step 7 of the Korevaar et al. approach [13].

#### Model structure

**Fig. 2:**
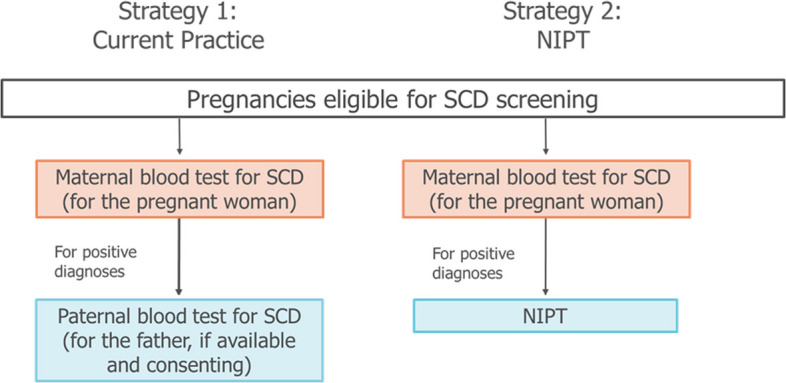
Outline of modelled strategies **Abbreviations**: NIPT: non-invasive prenatal testing; SCD: sickle cell disease

The analysis presented in this study employs a decision tree structure (built in Microsoft Excel) to deterministically model the impact of varying the sensitivity and specificity of NIPT on SCD diagnoses, to determine the MAC for a diagnostic study of NIPT as a potential replacement for the father testing stage of the current screening pathway (Fig. 2). Decision trees are appropriate for modelling the short-term time horizons when outcomes are based on well-defined processes, and this structure has further been used in other models of antenatal screening scenarios in a UK population [14–16].

The model starts at the point in which eligible pregnant women are offered a maternal carrier blood test as part of the SCD screening pathway. Pregnant women who decline the maternal carrier blood test, or the subsequent offer of NIPT in the respective strategy, are considered lost to follow-up, and pregnant women who receive a negative result from either the blood test or NIPT are not offered further testing.

In the current standard of care, the biological father is offered a paternal carrier blood test if the pregnant woman’s maternal carrier blood test result is positive; if both parents receive a positive carrier blood test result, or if there is no paternal carrier blood test result, PND is offered to diagnose the SCD status of the fetus. In the NIPT screening strategy, NIPT replaces the paternal carrier blood test: NIPT is offered to all women with a positive maternal carrier blood test result and, in the case of a high-chance NIPT result, invasive testing is offered to diagnose the SCD status of the fetus.

Invasive testing in the form of PND is not presented for the NIPT pathway, to avoid influencing the NIPT-specific MAC. NIPT results are expected to be reported before the offer of PND, and PND results are therefore not considered. This approach was adopted following discussions with the stakeholders during the first workshop, and later validated with stakeholders and in follow-up discussions with experts. The accompanying manuscript models the effectiveness of including NIPT in the screening pathway and includes results that are discussed in detail for the full pathway, including PND [12].

### Model inputs

Prevalence of SCD in the sickle cell and thalassaemia screening population, screening uptake, and screening performance were informed by the latest NHS Sickle Cell and Thalassaemia Screening Programme Data Report for England, which collected data for the period March 2019–April 2020. The model population is in line with the number of pregnant women offered SCD screening in England during this timeframe [8]. Where data were not available from the screening report, such as for the uptake of NIPT, targeted searches were conducted to source these inputs from the published literature. Expert opinion, obtained during the first workshop, was used to inform any remaining inputs. The full list of model inputs, their rationale for use in the model, and corresponding sources is presented in Table 1.

**Table 1:** Model inputs

Input	Model input	Rationale	Source
Pregnancies offered maternal carrier blood test	616573	Number of screening samples for SCD, thalassaemia and other haemoglobin variants in England (2019/20)	NHS SCT Screening Programme data report (2019/20) [8]
Proportion declining maternal carrier blood test	0.13%	Rate of declines for antenatal screening in England (2019/20)	NHS SCT Screening Programme data report (2019/20) [8]
Proportion of women being offered PND	0.74%	Based on number of identified high-risk fathers + number of screen positive pregnancies without father available	Expert advice obtained during workshop 1
Proportion of women being offered NIPT	2.13%	Screen positive results (% of maternal carrier blood test samples, 2019/20)	NHS SCT Screening Programme report (2019/20) [8]
Pregnancies at risk of SCD	47.42%	Proportion of screen-positive women for SCD following maternal carrier blood test (2019/20)	NHS SCT Screening Programme data report, additional information (2019/20) [8]
Pregnancies at risk of alpha thalassaemia	6.91%	Proportion of screen-positive women for alpha thalassaemia following maternal carrier blood test (2019/20)	NHS SCT Screening Programme data report, additional information (2019/20) [8]
Pregnancies at risk of beta thalassaemia	34.45%	Proportion of screen-positive women for beta thalassaemia following maternal carrier blood test (2019/20)	NHS SCT Screening Programme data report, additional information (2019/20) [8]
Proportion accepting NIPT	90.71%	Referenced in Taylor-Phillips 2015 as the assumed proportion who would accept the offer of cfDNA testing following a ‘high-risk’ combined result for T21, T18 and T13 testing in the UK	Taylor-Phillips 2015 [15]
Proportion declining NIPT	9.29%	Calculated as the proportion who would decline cfDNA testing, using the assumed proportion who would accept the offer of cfDNA testing following a ‘high-risk’ combined result for T21, T18 and T13 testing in the UK	Taylor-Phillips 2015 [15]
Failure rate of NIPT	8.00%	Number of NIPT which fail and require retesting	Expert advice obtained during workshop 1
Proportion of fetuses positive for SCD (overall population)	0.04%	Calculated as the proportion of fetuses which test positive for a significant condition (245) following newborn blood spot screening for SCD as a proportion of the total number of women who accept the initial maternal carrier blood test (616,573) in the year 2019/20	NHS SCT Screening Programme data report (2019/20) [8]
Proportion of fetuses positive for SCD (screen positive population)	3.94%	Calculated as the proportion of fetuses which test positive for a significant condition (245) among pregnant women who have received a positive maternal carrier blood test (6216) in the year 2019/20	NHS SCT Screening Programme data report (2019/20) [8]

Abbreviations: *NHS* National Health Service, *NIPT* non-invasive prenatal testing, *PND* prenatal diagnosis, *SCD* sickle cell disease, *SCT* sickle cell and thalassaemia.

Assumptions used in the study are listed in Table 2 and were validated by stakeholders to ensure that they were representative of what would likely be observed in clinical practice.

**Table 2:** Assumptions used in the model

Assumption	Justification/rationale
Positive predictive value of paternal carrier blood test, assumed to be 25%	Assumed to be 25%, as parents who are both carriers of the single mutation in the β-globin gene which causes SCD have a 1 in 4 chance of producing a child who then inherits both recessive genes
Screening options are available to all pregnancies regardless of gestational stage	To aid with simplicity of modelling, time-dependency was not included in the model
Paternal carrier testing is considered error-free in identifying carrier status	Assumed for simplicity of modelling, as current clinical practice regards paternal carrier testing as reliable

Abbreviations: *SCD* sickle cell disease.

### Model outcomes

The key model outcomes are the number of fetuses with true and false positive and negative diagnoses, which are used to identify the MAC for NIPT. In addition, the model tracks the number of declined tests at each step in the pathway.

### MAC identification

During the second workshop and in follow-up discussions with stakeholders, two main aspects were identified as important when defining the MAC:

- Positive predictive value (PPV), calculated as the proportion of fetuses with true positive diagnoses over the number of total fetuses with positive diagnoses following NIPT.
- Negative predictive value (NPV), calculated as the proportion of fetuses with true negative diagnoses over the number of total fetuses with negative diagnoses following NIPT.

Building on this, two scenarios are thus presented as alternative thresholds for the MAC of NIPT. In both scenarios, the positive predictive value would be equal to 25%, to match the proportion of pregnancies where both parents are SCD carriers (in situations of a completed testing protocol in the current screening pathway; see Table 2 and Appendix 1: Supplementary Fig. 1). These two MAC scenarios are outlined in more detail below:

- *Scenario 1*, in which the accuracy of NIPT should result in a maximum of 9 false negative diagnoses, as this was regarded by stakeholders as the absolute maximum number of false negative diagnoses which would be acceptable, given that it is predicted that in clinical practice, an increased number of women will be provided with more accurate risk information, therefore improving the accuracy of the overall programme.
- *Scenario 2*, in which the accuracy of NIPT should result in a maximum of 2 false negative diagnoses, which may be regarded as a more desirable threshold to be applied in clinical practice, while still being realistic.

### Sensitivity and specificity analyses

The sensitivity and specificity of NIPT were varied separately in 0.5% increments, in order to test the impact of NIPT accuracy on model outcomes and identify the levels of accuracy that would allow for meeting the respective scenario thresholds. The numbers of true positives and false negatives, and false positives and true negatives were recorded for each incremental variation of the sensitivity and specificity respectively.

### Sample size calculation

The determined sensitivity and specificity MAC for NIPT were then used to perform a sample size calculation. The formulae below, based on Korevaar et al. 2019 [13], were used to derive the required number of SCD-positive fetuses (based on the minimally acceptable value of sensitivity) and for required number of maternal SCD-negative fetuses (based on the minimally acceptable value of specificity):

$$\begin{array}{c}\text{D }= \frac{{{(Z}^{1-a*}\sqrt{Sen{s}_{0}\left(1-Sen{s}_{0}\right)}+{Z}^{1-\beta *}\sqrt{Sen{s}_{1} \left(1-Sen{s}_{1}\right))}}^{2}}{{\left(Sen{s}_{1}-Sen{s}_{0}\right)}^{2}}\\ ND={\frac{({Z}^{1-a*}\sqrt{Spe{c}_{0}\left(1-Spe{c}_{0}\right)}+{Z}^{1-\beta *}\sqrt{Spe{c}_{1}\left(1-Spe{c}_{1}\right))}}{{\left(Spe{c}_{1}-Spe{c}_{0} \right)}^{2}}}^{2}\end{array}$$

where

$$a*=1-\sqrt{1-a}$$

$a$ Represents rejection of a true null hypothesis (i.e., a false positive result)

$$\beta *=1-\sqrt{1- \beta }$$

$\beta$ Represents failure to reject a false null hypothesis (i.e., a false negative result)

$${Z}^{1-a*}= {\phi }^{-1}\left(1-a*\right)$$

$${Z}^{1-\beta *}= {\phi }^{-1}\left(1- \beta *\right)$$

- *D* is the required number of SCD-positive fetuses
- *ND* is the required number of SCD-negative fetuses
- *Sens*_*0*_ is the minimally acceptable value for sensitivity
- *Spec*_*0*_ is the minimally acceptable value for specificity
- *Sens*_*1*_ is the expected value of sensitivity
- *Spec*_*1*_ is the expected value of specificity

Probabilities for type 1 error (α; incorrect rejection of a true null hypothesis) and type 2 error (ß; incorrect failure to reject a false null hypothesis) were set to 5% and 10%, respectively (in line with the values applied for the sample calculations provided by Korevaar et al. 2019) [13]. The sensitivity and specificity were incrementally varied from the value obtained as the MAC for each parameter in 0.5% steps. The sample size calculations were started at a sensitivity and specificity 0.5% higher than those identified as the MAC in each scenario, in order to avoid denominator values of zero (due to subtracting the exact same minimally acceptable value from the corresponding expected value) resulting in erroneous calculations. Varying the sensitivity impacted on the required number of fetuses with SCD and varying the specificity impacted the required number of fetuses without SCD in the sample size for a hypothetical diagnostic test. Once obtained, the required number of fetuses with SCD was divided by the assumed prevalence of SCD among the fetuses within the population of pregnant women who have received a positive maternal carrier blood test, calculated as 3.94% (Table 1), to identify the overall sample size required within this group.

The outcomes of this calculation represent the hypothetical numbers of fetuses with and without SCD, and the overall number of women with an SCD screen-positive maternal carrier blood test result, required to draw a methodologically rigorous conclusion as to the lower limits of one-sided confidence intervals for sensitivity or specificity of NIPT and determine the MAC in a real-world setting.

## Results

### Minimally acceptable criteria

As described in the study approach, the MAC for NIPT were determined based on stakeholder workshops to achieve the thresholds for PPV and NPV. These thresholds were linked to two scenarios, with results described below. Both scenarios required a minimum PPV of 25%, aligning with the proportion of pregnancies where both parents are SCD carriers in the current screening pathway (see Table 2 and Appendix 1: Supplementary Fig. S1). The determination of this MAC ensured that even with false positive, clinical practice would include the offer of PND to confirm diagnoses, thereby refining the final outcomes. In both scenarios, the results directly reflect the stakeholder-defined MAC, ensuring that the clinical application of NIPT achieves the desired balance of sensitivity, specificity, and predictive values to meet the established thresholds outlined in the second workshop.

**Fig. 3:**
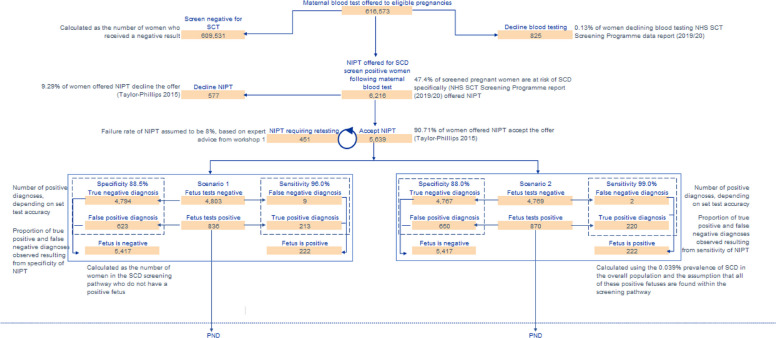
Pathway diagram for Scenario 1 and Scenario 2 in the model Abbreviations: *NHS* National Health Service, *NIPT* non-invasive prenatal testing, *SCD* sickle cell disease, *SCT* sickle cell and thalassemia

The breakdown of pregnancies included at each stage of the modelled pathway for Scenario 1 and Scenario 2 are presented in Fig. 3. The NIPT pathway diverges from the standard of care following the maternal carrier blood test. Following a positive maternal carrier blood test, 6216 pregnancies are estimated as at risk of SCD in England each year and are offered NIPT. Of these, 577 decline the offer and are regarded as lost to follow-up, with no further interventions modelled or outcomes reported. Conversely, 5639 women accept the offer of NIPT for SCD (90.71%); of these, 451 NIPT procedures fail and require retesting (8%). The outcomes used to inform the MAC for NIPT draw from the 5639 modelled women who accept the offer of NIPT and undergo a successful test, either at the first attempt or at re-testing.

#### Scenario 1

The MAC for NIPT in this scenario includes a maximum of 9 false negatives per year and a minimum PPV of 25%. These numbers were achieved with a sensitivity of 96.0% or greater, and a specificity of 88.5% or greater. The full results for the modelled MAC and outcomes for Scenario 1 are outlined in Table 3.

**Table 3:** Outcomes for Scenario 1 MAC

Outcome	Value
Sensitivity	96.0%
Specificity	88.5%
Accuracy ^a^	88.80%
PPV	25.51%
NPV	99.81%
Number of true negatives	4794
Number of false negatives	9
Number of true positives	213
Number of false positives	623

^a^Accuracy has been calculated as the number of true (negative and positive) diagnoses divided by the total number of diagnoses

The set sensitivity and specificity for NIPT translate to 4794 true negatives and 9 false negatives, leading to an NPV of 99.81% and 213 true positives, and 623 false positives, leading to a PPV of 25.51%. Although this PPV implies a significant proportion of false positive results, it is important to note that, in clinical practice, NIPT would be followed by the offer of PND. Furthermore, this PPV aligns with stakeholder-defined thresholds to provide improved risk information for women who do not have access to a paternal carrier blood test result and therefore enhance the accuracy of the overall program. The overall accuracy (proportion of true positives and negatives in all diagnoses) resulting from the sensitivity and specificity MAC is 88.80%.

#### Scenario 2

The MAC for NIPT in this scenario included a maximum of 2 false negatives per year and a minimum PPV of 25%. These values were achieved with a sensitivity of 99.0% or greater and a specificity of 88.0% or greater. The full results for the modelled MAC and outcomes are outlined in Table 4.

**Table 4:** Outcomes for Scenario 2 MAC

Outcome	Value
Sensitivity	99.0%
Specificity	88.0%
Accuracy ^a^	88.43%
PPV	25.29%
NPV	99.95%
Number of true negatives	4767
Number of false negatives	2
Number of true positives	220
Number of false positives	650

^a^ Accuracy has been calculated as the number of true (negative and positive) diagnoses divided by the total number of diagnoses

The set sensitivity and specificity for NIPT translate to 4767 true negatives and 2 false negatives, leading to an NPV of 99.95% and 220 true positives, and 650 false positives, leading to a PPV of 25.29%. The overall accuracy resulting from the sensitivity and specificity MAC is 88.43%.

### Sample size calculations

#### Scenario 1

The resulting sample size required to illustrate a one-sided confidence interval in a diagnostic study of NIPT with a MAC sensitivity of 96% and an expected sensitivity of 96.5% would be a minimum of 12,448 fetuses with SCD. Utilising an expected prevalence of SCD of 3.94%, the required sample size would be 315,824 overall pregnancies. Varying the expected sensitivity up to 100%, the number of pregnancies required would be 65 SCD-positive fetuses, and 1649 overall pregnancies.

With a minimally required specificity of 88.5% and an expected specificity of 89.0%, the resulting sample size required to illustrate a one-sided confidence interval for specificity would be a minimum of 34,279 fetuses without SCD. As the prevalence of SCD is assumed to be 3.94%, the prevalence of fetuses without SCD would be 96.1%. Utilising this percentage leads to 35,686 overall pregnancies necessary to estimate whether the expected specificity exceeds the MAC with sufficient precision. Varying the expected specificity up to 98.5%, the number of pregnancies required would be 47 SCD-negative fetuses, and 49 overall pregnancies.

The full results for the sample size calculations for incremental values of expected sensitivity and specificity are presented in Tables 5 and 6, respectively.

**Table 5:** Sample size calculations employing Scenario 1 MAC sensitivity

Expected sensitivity	Number of SCD-positive fetuses required	Overall sample size^a^
96.0%	N/A (since minimally acceptable sensitivity)	
96.5%	12448	315,824
97.0%	2927	74,262
97.5%	1213	30,776
98.0%	630	15,984
98.5%	366	9286
99.0%	225	**5709**
99.5%	140	**3552**
100.0%	65	**1649**

^a^ Overall sample size has been calculated by dividing the number of affected pregnancies required by the assumed prevalence of SCD in the relevant screening population (3.94%). Overall sample size numbers that are below the assumed size of the SCD-relevant screening population (6216 pregnancies) are highlighted in **bold**

**Table 6:** Sample size calculations employing Scenario 1 MAC specificity

Expected specificity	Number of SCD-negative fetuses required	Overall sample size^a^
88.5%	N/A (since minimally acceptable specificity)	
89.0%	34279	35,686
89.5%	8421	8767
90.0%	3675	**3826**
90.5%	2028	**2111**
91.0%	1272	**1324**
91.5%	865	**900**
92.0%	622	**648**
92.5%	465	**484**
93.0%	359	**374**
93.5%	283	**295**
94.0%	228	**237**
94.5%	186	**194**
95.0%	154	**160**
95.5%	128	**133**
96.0%	108	**112**
96.5%	91	**95**
97.0%	77	**80**
97.5%	65	**68**
98.0%	55	**57**
98.5%	47	**49**

^a^ Overall sample size has been calculated by dividing the number of non-affected pregnancies required by “1—the assumed prevalence of SCD in the relevant screening population” (96.06%). Overall sample size numbers that are below the assumed size of the SCD-relevant screening population (6216 pregnancies) are highlighted in **bold**

#### Scenario 2

With a minimally acceptable sensitivity of 99.0% and an expected sensitivity of 99.5%, the resulting sample size required to illustrate a one-sided confidence interval in a diagnostic study of NIPT would be a minimum of 2582 SCD-positive fetuses, and 65,509 overall pregnancies. Varying the expected sensitivity up to 100%, the number of pregnancies required would be 268 SCD-positive fetuses, and 6800 overall pregnancies.

With a minimally acceptable specificity of 88.0% and an expected specificity of 88.5%, the resulting sample size required to illustrate a one-sided confidence interval in a diagnostic study of NIPT would be a minimum of 35,597 SCD-negative fetuses, and 37,058 overall pregnancies. Varying the expected specificity up to 98.0%, the number of pregnancies required would be 51 SCD-negative fetuses, and 53 overall pregnancies.

The full results for the sample size calculations for incremental values of sensitivity and specificity are presented in Tables 7 and 8, respectively.

**Table 7:** Sample size calculations employing Scenario 2 MAC sensitivity

**Expected Sensitivity**	Number of SCD-positive fetuses required	Overall sample size^a^
99.0%	N/A (since minimally acceptable sensitivity)	
99.5%	2582	65,509
100.0%	268	6800

^a^Overall sample size has been calculated by dividing the number of affected pregnancies required by the assumed prevalence of SCD in the relevant screening population (3.94%). Overall sample size numbers that are below the assumed size of the SCD-relevant screening population (6216 pregnancies) are highlighted in **bold**

**Table 8:** Sample size calculations employing Scenario 2 MAC specificity

**Expected Specificity**	Number of SCD-negative fetuses required	Overall sample size^a^
88.0%	N/A (since minimally acceptable specificity)	
88.5%	35,597	37,058
89.0%	8752	9111
89.5%	3823	**3980**
90.0%	2112	**2199**
90.5%	1326	**1380**
91.0%	903	**940**
91.5%	650	**677**
92.0%	487	**507**
92.5%	376	**391**
93.0%	297	**309**
93.5%	240	**250**
94.0%	196	**204**
94.5%	162	**169**
95.0%	136	**142**
95.5%	114	**119**
96.0%	97	**101**
96.5%	83	**86**
97.0%	71	**74**
97.5%	60	**62**
98.0%	51	**53**

^a^ Overall sample size has been calculated by dividing the number of non-affected pregnancies required by “1—the assumed prevalence of SCD in the relevant screening population” (96.04%). Overall sample size numbers that are below the assumed size of the SCD-relevant screening population (6216 pregnancies) are highlighted in **bold**

## Discussion

### MAC and sample size results

Our study explores two scenarios for defining the MAC of NIPT, to inform the sample size of a future diagnostic accuracy study evaluating the accuracy of NIPT in replacing the paternal carrier blood test in the current SCD screening pathway. Diagnostic studies play an important role in determining the impact of implementing a new test. However, these studies often do not pre-specify a study hypothesis, raising questions about the rigour of their methodology and robustness of conclusions [13, 17]. Within SCD specifically, the importance of undertaking a methodologically rigorous diagnostic study for a new screening strategy cannot be understated. The implementation of a novel test which is diagnostically inferior to the current pathway may result in women receiving incorrect information about their pregnancy and undergoing unnecessary procedures, some of which may be invasive in nature.

It should be noted that the sensitivity and specificity for NIPT identified in each scenario presented in this study are lower than other similar applications of NIPT testing in a screening context. Where NIPT is applied to trisomy testing, the accuracy has been shown to be more precise than the standard of care combined screening test within the NHS Fetal Anomaly Screening Programme – cfDNA testing for trisomy 21 had higher sensitivity, a lower false positive rate and higher PPV [18, 19]. While in the context of fetal anomaly screening NIPT has been shown to be more effective than the standard of care, this has not been evaluated for the SCD programme. As a result, the MAC and subsequent outcomes presented for NIPT in each scenario in this study are intended to at least match the performance of the current paternal carrier blood test stage of the SCD screening pathway.

The PPV and NPV are key outcomes of interest for any screening test. The high number of false positive diagnoses following NIPT in both scenarios (as modelled in this study) would be in the group offered follow-up PND in practice and is equivalent to the proportion of non-affected fetuses diagnosed by the current screening programme. Therefore, while the MAC presented in both scenarios meet the hypothetical PPV of the completed paternal carrier blood testing stage of the current screening pathway, it is a minimum and a pre-PND probability of 25% is the current standard. A PPV of this level provides more accurate risk information for screen-positive women with no paternal blood test result who remain eligible for PND in the standard of care pathway. However, the introduction of NIPT may lead to an increase in uptake of PND following a false positive result, and future diagnostic accuracy studies may employ a more stringent PPV threshold if it is achievable.

With regards to the NPV, the modelled placement of NIPT in the screening pathway presents the need to ensure that as few pregnant women as possible receive false negative results following NIPT, because no further testing would be offered to them in the antenatal period. In this situation, a pregnant woman undergoing SCD screening would be presented with incorrect information on which to make an informed decision as to the future of her pregnancy. As such, while Scenario 1 may present the most “minimally acceptable” number of false negative diagnoses to be observed following NIPT, real-world implementation of NIPT may strive to employ a MAC similar to Scenario 2, resulting in two, or fewer, false negative diagnoses.

Identifying the sample size required for a diagnostic study presents an important methodological step in determining whether a test will be effective in clinical practice. Bachmann et al. illustrate that often studies do not place an emphasis on determining the required sample size before a study begins, impacting on the robustness of conclusions obtained [20]. Approaches to calculating the sample size required for a diagnostic study are varied, but in situations where the aim of the study will be disease detection, it is as important to calculate the number of disease-positive participants as it is to calculate the overall number of participants following initial disease screening required to demonstrate diagnostic efficacy [21].

Within this study, it is noted that the sample size presented for the MAC in each scenario is unfeasible if the study design of choice, a cohort study in which all participants undergo the index test and the reference standard, is being considered; the number of SCD-positive fetuses, and overall sample size of pregnant women who have received an SCD-positive maternal carrier blood test result is higher than the number observed in England in a given year. There are different approaches to remedying the unrealistic sample size presented for these scenarios. If preliminary studies indicate that a sensitivity and specificity which are higher than the MAC are likely [10], one approach would be to increase the expected sensitivity and specificity for each scenario to a point where the number of SCD-positive fetuses and overall sample size approximates more closely to the number of observed positive diagnoses in the annual SCD screening programme—in Scenario 1, this would result in an expected sensitivity of 99.0% and an sample size requiring 225 SCD-positive fetuses and therefore an overall sample size of 5709 pregnant women. An expected specificity of 90.0% would require an overall sample size of 3826 pregnant women; in Scenario 2, an expected specificity of 89.5% would require an overall sample size of 3980 pregnant women, and increasing the sensitivity to 100% would produce an overall sample size requiring 268 SCD-positive fetuses and an overall sample size of 6800 pregnant women, which is greater than the number of women who have received an SCD-positive maternal carrier blood test result in England in 2019/20.

In the context of this study, increasing the expected sensitivity and specificity of NIPT to obtain a more realistic sample size would also change the PPV and NPV of each scenario. A second option might be to explore different statistical assumptions for the applied sample size calculations (with the calculated sample size being correlated to the assumed probability of Type 2 errors, for example) or the use of different study designs which have been proposed for low prevalence settings [22, 23]. The number of positive cases required to achieve statistical significance, the application of the index test in a large number of screen positive women and the application of invasive PND as the reference standard in a large number of women with negative NIPT results present particular challenges to the traditional cohort design in this setting. In these circumstances newborn screening for SCD might be considered a proxy reference standard and consideration might be given to the proposed “nested case–control” design as a means of reducing the number of index tests required. In addition, the ‘two phase’ design might be considered as a means of reducing the number of PNDs required. Both designs can support an analysis of population metrics such as PPV and NPV which have been identified as key outcomes by stakeholders. To address the practicalities of study requirements, a combination of these approaches may be needed.

However, even with these designs, and given the low prevalence of SCD in screen positive women, the number of cases required remains challenging for a single study. For this reason, a pragmatic, stepwise approach may also be useful. This might include the use of formal test accuracy studies which, depending on outcomes, give way to studies which are primarily geared to explore clinical utility outcomes but are also able to report accuracy outcomes in the longer term. The availability of newborn screening to provide a proxy reference standard which is currently delivered in routine practice may make such an approach possible. Finally, pooling outcomes in meta-analysis might be considered as studies of comparable NIPT methods in screen positive women become available.

Alternatively, other approaches to sample size which are less stringent than Korevaar et al. might be justified in low prevalence settings and should therefore be considered [22].

### Strengths and limitations

It is noted that the implementation of a new technology into an existing screening pathway requires more than an assessment of just the efficacy of the new technology, an aspect of which it outlined as part of step 2 of the Korevaar et al. process [13]. Other outcomes, notably the cost of the new technology, and specific to the SCD screening pathway, the number of PND diagnoses undertaken versus the standard of care are important dimensions which need to be evaluated when determining whether NIPT should be implemented. While these outcomes are not discussed in the present analysis, so as not to influence the MAC presented for NIPT in each scenario, these have been reported elsewhere, in order to provide a holistic overview of NIPT within the wider SCT screening pathway [12].

Utilising the approach outlined in Korevaar et al. provided a robust framework to guide the present analysis [13]. Due to the relatively novel use of cfDNA testing in a screening context, key stakeholders from the SCD screening programme and field of cfDNA research provided guidance during workshops throughout the study. One key role of these stakeholders was to validate the inputs used in the model, including any assumptions made in lieu of missing data, or to simplify the modelling process, outlined in Tables 1 and 2.

Obtaining stakeholder input at various points of the project, and in particular during the first and second workshops, provides additional strength and robustness to the study approach. Guidance from stakeholders was used to identify the issue of attendance at the paternal testing stage, which may not have otherwise been identified through literature, providing a real-world context to this study, and clear applicability to the screening pathway to be evaluated in a diagnostic study. Discussions with stakeholders assisted with the main aspects to be determined when identifying the MAC, and in particular weighing the consequence of test misclassifications, and specifically about the relative importance of PPV in the screening pathway. Furthermore, validating the inputs sourced from literature and assumptions to be used in the model with stakeholders during workshop one increased confidence that they would be representative of what would be observed in clinical practice, providing further robustness to the model and outcomes.

Despite the stakeholder validation, the assumptions made represent a limitation of the study. In assuming no time dependency in the model (i.e., all screening options available to all pregnancies regardless of gestational stage), the number of women who undertake NIPT is likely to be higher in the modelled population than would likely be observed in a real-world scenario. This is because data has shown that pregnant women are less likely to accept SCD screening as the pregnancy develops, although this change in screening uptake as the pregnancy progresses may be negated by the non-invasive nature of NIPT versus the current standard of care [8]. Furthermore, inputs sourced from published literature, such as the assumption referenced from Taylor-Phillips et al. that 90.7% of women will accept the offer of a non-invasive form of screening over an invasive method, highlight areas for future large-scale research in real-world conditions [15].

The Korevaar et al. approach was used in this analysis as it was considered more comprehensive than other methodologies used to determine the MAC and/or sample size for future diagnostic studies [13]. For instance, the approach outlined by Haijan-Tilaki presents a method of calculating the sample size, but overlooks the preliminary steps required to determine the MAC, presenting only one aspect of a wider process [24]. Korevaar et al. presented a novel and robust approach, due to its focus on the process of determining the sample size for a diagnostic study, including the addition of identifying the role of the test in the screening pathway and quantifying key outcomes to determine the MAC [13]. With this in mind, the step-wise approach presented by Korevaar et al. may somewhat oversimplify the process of identifying the MAC, particularly when defining the role of a new index test, and key outcomes of interest within a well-established screening pathway, such as the SCD screening pathway in England. Within the present analysis, evolving discussions with stakeholders as to the role of NIPT in the screening pathway meant that the initial steps of the Korevaar et al. approach were revisited. This, in turn, affected the key outcomes of interest when identifying the MAC; for instance, if NIPD were to be modelled as a replacement to PND, which is offered as a final stage of the current SCD screening pathway in England, identifying the MAC would place an increased emphasis on the number of false positives diagnosed and subsequent PPV as a key outcome of interest. As such, within a screening context, identifying the role of the new test to be used may be more of an evolving discussion throughout the analysis, rather than an initial stage in a linear process as is outlined in the Korevaar et al. approach.

## Conclusion

Based on the number of pregnancies at risk of SCD in the UK and at the agreed MAC, a traditional cohort test accuracy study is unachievable. A number of options have been proposed. Firstly, the sample size calculation could be based on increased sensitivity and specificity exceeding the MAC if this is likely to be achievable based on preliminary studies. Secondly, study design mitigations based on guidance for test accuracy studies in low prevalence settings should be considered. Studies in UK populations are ongoing and discussion of these options should continue in light of their results. In the longer term, consideration of the appropriate approach to sample size calculation in low prevalence settings should be considered. In addition to a diagnostic study, the utilisation of NIPT in the SCD screening pathway would benefit from further studies (such as further qualitative research on the real-world uptake of NIPT in an SCD screening context) in order to provide more robust data to better inform decisions on the future implementation of NIPT.

## Supplementary Information

Supplementary Material 1.

## Data Availability

Data is provided within the manuscript, or taken from published data with references in the text.

## Abbreviations

CE: Capillary electrophoresis
cfDNA: Cell-free deoxyribonucleic acid
HPLC: High performance liquid chromatography
MAC: Minimally acceptable criteria
NHS: National Health Service
NIPD: Non-invasive prenatal diagnosis
NIPT: Non-invasive prenatal testing
NPV: Negative predictive value
NSC: National Screening Committee
PND: Prenatal diagnoses
PPV: Positive predictive value
SCD: Sickle cell disease
UK: United Kingdom
