# Supplementary material for: Minimally acceptable criteria and required sample size for an accuracy study of non-invasive prenatal testing of sickle cell disease in screen positive women in England: results of a decision tree model
Source: Diagn Progn Res. 2026 Jun 15;10:18. doi: 10.1186/s41512-025-00192-w (PMC13267277; doi:10.1186/s41512-025-00192-w)
Supplement: Supplementary file 1 — Supplementary Material 1. [file 41512_2025_192_MOESM1_ESM.docx]

Appendix

Supplementary Figure S1. Outline of the completed father testing protocol

Abbreviations: SCD: sickle cell disease.
